# Supplementary material for: Prevention of 7-Ketocholesterol-Induced Overproduction of Reactive Oxygen Species, Mitochondrial Dysfunction and Cell Death with Major Nutrients (Polyphenols, ω3 and ω9 Unsaturated Fatty Acids) of the Mediterranean Diet on N2a Neuronal Cells
Source: Molecules. 2020 May 13;25(10):2296. doi: 10.3390/molecules25102296 (PMC7287847; doi:10.3390/molecules25102296)
Supplement: Supplementary file 1 [file molecules-25-02296-s001.pdf]

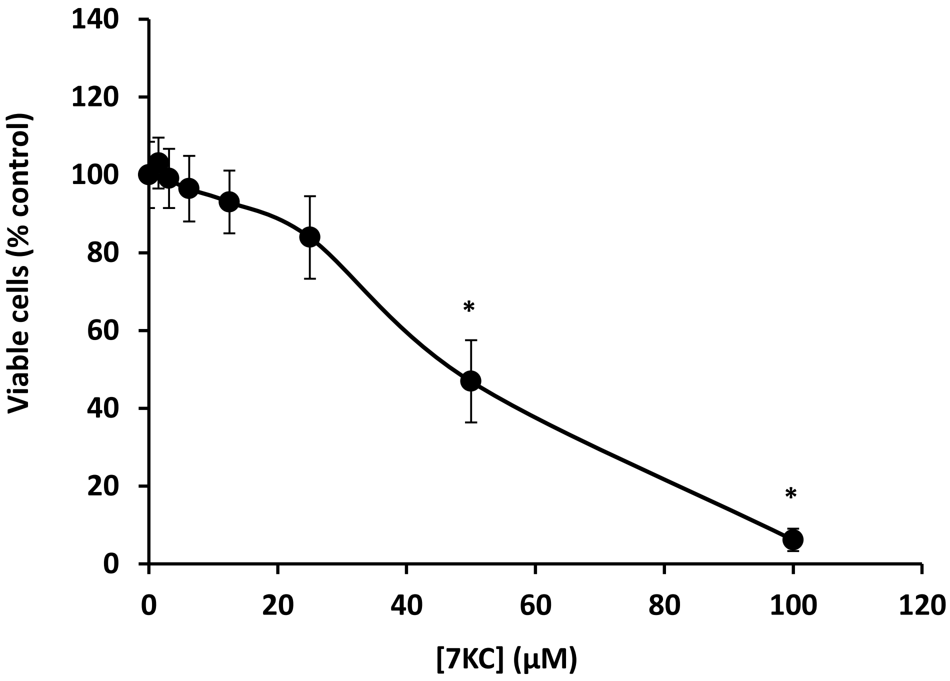


**Figure S1.** Evaluation with the fluorescein diacetate (FDA) assay of the effects of 7-ketocholesterol on the cell viability of N2a cells. Murine neuroblastoma N2a cells, previously cultured for 24 h, were further cultured for 48 h with or without 7-ketocholesterol (7KC) at concentrations ranging from 1.5 to 100 µM. The results are in percentages relatively to the control (untreated cells). Data obtained with the FDA are shown. Data shown are expressed as mean ± standard deviation (SD) of four independent experiments performed in triplicate. Significance of the differences between control (untreated cells) and 7KC-treated cells; Mann Whitney test: * $P<0.05$ or less.
